# Supplementary figures and images for: Exploring factors affecting the adoption and continuance usage of drone in healthcare: The role of the environment
Source: PLOS Digit Health. 2023 Nov 7;2(11):e0000266. doi: 10.1371/journal.pdig.0000266 (PMC10629621; doi:10.1371/journal.pdig.0000266)

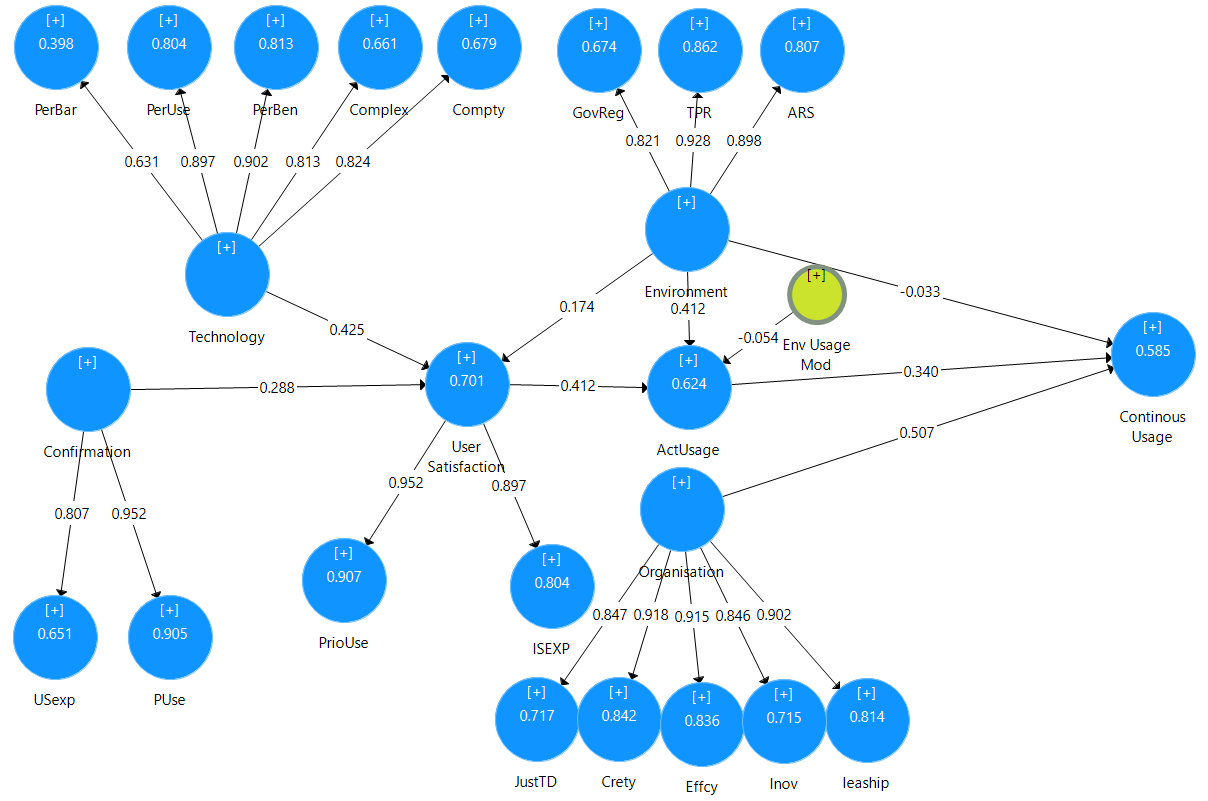

Supplement: S1 Fig — (PNG) [file pdig.0000266.s003.png]
