# Supplementary material for: Exploring factors affecting the adoption and continuance usage of drone in healthcare: The role of the environment
Source: PLOS Digit Health. 2023 Nov 7;2(11):e0000266. doi: 10.1371/journal.pdig.0000266 (PMC10629621; doi:10.1371/journal.pdig.0000266)
Supplement: Attachment — Submitted filename: Response to Reviewers.docx [file pdig.0000266.s004.docx]

**Response to Comments from Editors and Reviewers**

**Comments:**

1. Does this manuscript meet PLOS Digital Health’s publication criteria? Is the manuscript technically sound, and do the data support the conclusions? The manuscript must describe methodologically and ethically rigorous research with conclusions that are appropriately drawn based on the data presented.

Reviewer #1: Partly

Reviewer #2: Yes

***Response to :***

***Reviewer #1***: Thank you very much for your response. We have duly made modifications in the manuscript according to the recommendations and suggestions from the reviewers. We believe that, the changes applied meets PLOS Digital Health’s publication criteria.

***Reviewer #2***: Thank you very much for your response. We are grateful for this comment.

2. Has the statistical analysis been performed appropriately and rigorously?

Reviewer #1: I don't know

Reviewer #2: Yes

***Response to :***

***Reviewer #1***: Thank you very much for your response. We leveraged Smart PLS, a variance-based structural equation modelling approach for the data validation and analysis. We have provided all output of the analysis in the re-submission.

***Reviewer #2***: Thank you very much for your response. We are grateful for this comment.

3. Have the authors made all data underlying the findings in their manuscript fully available (please refer to the Data Availability Statement at the start of the manuscript PDF file)?

The PLOS Data policy requires authors to make all data underlying the findings described in their manuscript fully available without restriction,

with rare exception. The data should be provided as part of the manuscript or its supporting information, or deposited to a public repository. Forexample, in addition to summary statistics, the data points behind means, medians and variance measures should be available. If there are

restrictions on publicly sharing data—e.g. participant privacy or use of data from a third party—those must be specified.

Reviewer #1: No

Reviewer #2: Yes

***Response to :***

***Reviewer #1***: Thank you very much for your response. We have included all output of the analysis in the re-submission.

***Reviewer #2***: Thank you very much for your response. We are grateful for this comment.

4. Is the manuscript presented in an intelligible fashion and written in standard English?

PLOS Digital Health does not copyedit accepted manuscripts, so the language in submitted articles must be clear, correct, and unambiguous. Any

typographical or grammatical errors should be corrected at revision, so please note any specific errors here.

Reviewer #1: Yes

Reviewer #2: Yes

***Response to :***

***Reviewer #1 and #2***: Thank you very much for your response. We are grateful for this comment.

5. Review Comments to the Author

Reviewer #1: This paper seeks to explore the role of environment in the usage and continued usage of drones to deliver healthcare supplies inGhana. This provides an interesting overview, but there are some problems with the current manuscript. In particular the authors posit that this is a survey of drone users, At the beginning of the section 3.2.2, it states that “The participants of the study included only staff who make use of drugs and health commodities delivered through drones but 76% of the respondents had not experienced drone delivery according to the demographics. The authors should explain this and how it impacts their analysis.

***Response to reviewer comment***:

Thank you very much for your response. The study was conducted at the firm level. Participants of the study were individuals who worked with the health facilities. The question asked was "Have you ever experienced the operations of drones in healthcare delivery?" We sought to understand from the individual participants whether they have personally experienced drone delivery in their health facilities. The question was not in relation to the health facility as a whole but to the respondent as an agent or employee of the health facility. As it turned out, while the health facilities have experienced drone delivery services, some of the staff have not directly experienced drone deliveries.

Section 2.4 Need references to theories being used in the theoretical framework. The ECT is a theory to explain purchase and use, but who are the users in this context – is this a survey of the health institutes or the patients receiving the care? "ECT has a limitation in explaining the information system expectation formation process" (Yang, 2017). – what is this limitation and how is it relevant.

***Response to reviewer comment***: Thank you for this response. We have included references to the ECT and TOE theories as used in the theoretical framework. Secondly, the study is a survey of health facilities. The term "users" as used in the article refers to health facilities. Finally, one limitation of ECT in explaining the information system expectation formation process is that it primarily focuses on the individual level of analysis and may overlook the social and contextual factors that influence expectations. In the context of information systems, expectations are often shaped by factors such as social influence, cultural norms, organizational context, and technological advancements. ECT does not adequately capture these external influences on expectation formation. This limitation contributed to the introduction of the TOE theory to explain technology adoption in organizations and describe the process of its adoption and implementation.

Questions table

Some of the phrasing of questions is problematic as there are multiple decisions to be made within single question, this makes it difficult to understand the exact nature of influence.It would be useful to see the full factor mappings table, as I am sure some items also map on to other constructs. The way decisions were made as to which factor they belonged to should be explained. The construct of organisation creativity needs more explanation as to its relevance to this work, Unclear how the individual factors are used to calculate the constructs, eg environment construct is made up of multiple factors, how do they relate to each other, and why this particular focus?

***Response to reviewer comment***: Thank you very much for this comment. We have provided the full factor mappings table in the article. We used a variance-based structural equation modeling approach with SmartPLS for the model estimation and assessment as indicated in section 4.2 of our work. The technology, confirmation, user satisfaction, organization, and environment are all second-order constructs. We used formative approach in SmartPLS to map the first-order constructs to the second-order constructs. The construct of organization creativity is relevant to this work because it involves assessing the perception and belief of participants regarding the role of creativity within health facilities that use drone delivery services. The construct is measured through four specific questions that (see Table 2: Measures assessment results) capture different aspects of how creativity is valued and perceived within these organizations.

Results

The paper states that Convergent validity is measured against alternative measures – but what are these measures?

***Response to reviewer comment***:Convergent validity is evaluated by examining whether the scores obtained from the measure under investigation correlate strongly with scores from other established measures that theoretically measure the same or similar constructs (threshold of .50) (Fornell & Larcker, 1981). High correlations between the measures indicate that they are converging or converging validity is established. The convergent validity test was carried out by accessing the resulting values of the Average Variance Extracted (AVE). The results showed that all the measures had their AVE statistics higher than the recommended threshold of .50 indicating that all the measures are adequately valid. Convergent validity test was carried out using the SmartPLS software

**Reviewer #2**: This is a nice body of evidence which will add to the emerging tech space . Authors elaborated a lot in explaining the concepts and methodology, suggest to elaborate more on results and bring in more substance into the discussion. Make the literature search and concept description short.

***Response to reviewer comment***:Thank you very much for your comment. We have elaborated more on the results and shortened the literature concepts.

6. PLOS authors have the option to publish the peer review history of their article (what does this mean?). If published, this will include your full peer review and any attached files.

**Do you want your identity to be public for this peer review?** If you choose “no”, your identity will remain anonymous but your review may still be made public. For information about this choice, including consent withdrawal, please see our Privacy Policy.

Reviewer #1: No

Reviewer #2: Yes: Shibu Vijayan
